# MicroRNA-125a influences breast cancer stem cells by targeting leukemia inhibitory factor receptor which regulates the hippo signaling pathway

**DOI:** 10.18632/oncotarget.3953

**Published:** 2015-04-29

**Authors:** Sushmita Bose Nandy, Arunkumar Arumugam, Ramadevi Subramani, Diego Pedroza, Keziah Hernandez, Edward Saltzstein, Rajkumar Lakshmanaswamy

**Affiliations:** ^1^ Center of Excellence in Cancer Research, Department of Biomedical Sciences MSB1, El Paso, Texas, USA; ^2^ Graduate School of Biomedical Sciences, El Paso, Texas, USA; ^3^ Sadie and Annabelle Garbar Breast Care Center, Texas Tech University Health Sciences Center, Paul L. Foster School of Medicine, El Paso, Texas, USA

**Keywords:** breast cancer, stem cells, miR-125a, LIFR, hippo signaling

## Abstract

Cancer stem cells (CSC) are the main driving force behind cancer initiation and progression. The molecular mechanisms that regulate CSC properties are poorly understood. MicroRNAs (miRNAs) play a significant role in normal and cancer tissues. Here, we show that miRNA-125a indirectly regulates TAZ, an effector molecule in the Hippo pathway, through the leukemia inhibitory factor receptor (LIFR). The miR-125a→LIFR axis affected the homeostasis of nonmalignant and malignant breast epithelial stem cells through the Hippo signaling pathway. Inhibition of miR-125a in breast cancer cells led to a significant reduction in the CSC pool. In contrast, enhanced expression of miR-125a in nonmalignant breast epithelial cells resulted in significant expansion of the stem cell pool. Gain of function and loss of function of LIFR directly correlated with the inhibition and overexpression of miR-125a, respectively. Modulation of miR-125a led to a change in the activity of TAZ and its subcellular localization. We further demonstrated that miR-125a influenced stem cells by regulating Hippo signaling through LIFR in human primary breast cancer cells confirming the data obtained from established cell lines. We suggest that miR-125a could be a potential target against CSCs that maybe used along with the existing conventional therapies.

## INTRODUCTION

Chemoresistance and recurrence of cancers are attributed to a small population of cells within the cancers, designated as cancer stem cells (CSCs) [1-2]. Currently, knowledge about CSCs is lacking, and present understanding of the molecular and cellular mechanisms governing breast and other cancers is incomplete. MicroRNAs are negative regulators of genes, which repress expression at the post-transcriptional level. They regulate various properties of CSCs, including self-renewal, differentiation, proliferation, and fate determination, by affecting several key signaling pathways at the molecular level [3]. MiR-125a was reported to have diverse functions in normal as well as in malignant conditions [4]. However, its role in breast epithelial stem cells is currently unknown. In our previous studies of breast cancer occurrence, we found that miR-125a was upregulated in high risk breast epithelial stem cells compared to low risk groups [5]. Conversely, in the same study, an inverse expression of leukemia inhibitory factor receptor (LIFR) in relation to miR-125a was observed. LIFR is a known tumor suppressor in breast cancer and an upstream regulator of Hippo signaling [6]. Dysregulation of the phosphorylation status of Hippo signaling effectors, especially TAZ, has been associated with enhanced self-renewal and proliferative capabilities of stem cells, leading to a pro-carcinogenic environment [7-8]. These findings prompted us to examine the role of miR-125a in malignant breast epithelial stem cells, and determine its influence on Hippo signaling by altering LIFR expression.

Here, we have investigated non-malignant and malignant breast epithelial stem cells to determine the 1) role of miR-125a as a repressor of LIFR, and 2) impact of the miR-125a→LIFR axis on stem cell functions. We found that miR-125a regulated stem cell pool homeostasis. In addition, we also provided evidence that miR-125a targeted LIFR, and as a result, influenced the activity of the Hippo signaling effector molecule TAZ. We also showed that LIFR modulation replicated the effects of miR-125a on the stem cell pool. Our data strongly suggested that miR-125a could be a potential therapeutic target against CSCs.

## RESULTS

### Cancer stem cells express higher levels of miR-125a

**Figure 1:**
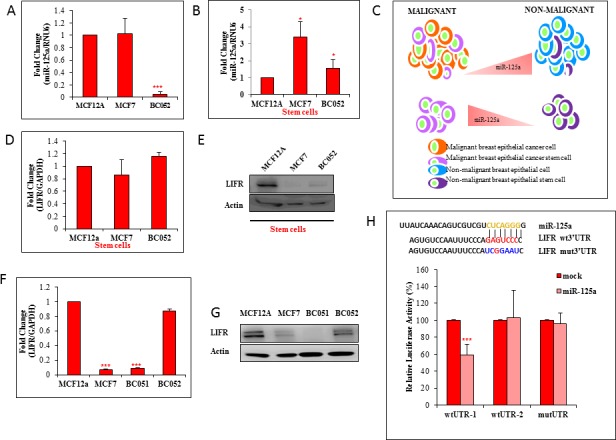
Inverse correlation between miR-125a and LIFR in stem cells **A.** qRT-PCR data shows expression of miR-125a in MCF7 and human primary breast cancer cells (BC-052) compared to MCF12A cells **B.** Expression levels of miR-125a in MCF7 and primary breast CSCs as compared to MCF12A stem cells. **C.** Pictorial representation of the relative levels of miR-125a in malignant and non-malignant stem cells and bulk cells **D.** Transcript level of LIFR in MCF7 and human primary breast cancer stem cells as compared to MCF12A stem cells. **E.** Immunoblotting analysis of LIFR in MCF7 and human primary breast cancer stem cells as compared to MCF12A stem cells. **F.** Transcript level of LIFR in MCF7 and human primary breast cancer cells as compared to MCF12A cells **G.** Immunoblotting analysis of LIFR in MCF7 and primary breast cancer cells as compared to MCF12A cells. **H.** Predicted interaction of miR-125a with LIFR *in silico*. Luciferase assay data for HEK293 cells demonstrating a decrease in the luciferase activity with addition of miR-125a mimics to wtLIFR 3′UTR-1 expressing cells but not in wtLIFR 3′UTR-2 and mutLIFR 3′UTR.

With the aim to determine the basal levels of miR-125a in cancer and normal breast epithelial cells, we used malignant MCF7, primary breast cancer (BC052 and BC051) and non-malignant MCF12A cells. We also used human normal breast tissue and breast cancer tissue. Quantitative real time RT-PCR revealed a decreased transcript level of miR-125a in primary breast cancer cells (0.95 fold), but apparently no change in MCF7 cells compared to nonmalignant MCF12A cells (Figure 1A). Transcript levels of miR-125a in human breast cancer tissues were found to be downregulated compared to normal breast tissues by 1.75 fold (Figure S1A). We next determined the basal expression levels of miR-125a transcripts in stem cells obtained from MCF12A, MCF7, and primary breast cancer cells. An increased transcript level of miR-125a, by 3.41 and 1.53 fold was observed in MCF7 and primary breast cancer stem cells, respectively, compared to MCF12A stem cells (Figure 1B). In addition, the transcript levels of miR-125a in a cell population devoid of stem cells (ALDH-), in MCF12A and MCF7 cells, showed no difference in expression levels (Figure S2A). Our findings provided a clear indication that the baseline expression levels of miR-125a in stem cells was different than in the normal cell population (Figure 1C).

### LIFR is a target of miR-125a

In one of our previous studies using a rat model, we showed a downregulation of LIFR expression in mammary epithelial stem cells from a high risk group (nulliparous) compared to a low risk group (parous) for breast cancer occurrence [5]. Interestingly, *in silico* analysis revealed LIFR to be a putative target for miR-125a. LIFR has been identified as a tumor suppressor in breast cancer [6, 9]. In lieu of this, we further wanted to investigate whether LIFR was regulated by miR-125a. The basal transcript expression level of LIFR in MCF7 and primary breast cancer stem cells was similar to MCF12A stem cells (Figure 1D). However, LIFR protein was downregulated in both MCF7 and primary breast stem cells compared to MCF12A stem cells, thus demonstrating an inverse pattern of expression between miR-125a and LIFR (Figure 1E). However, inverse relationship between miR-125a and LIFR were observed in other breast cancer cell lines also (Figure S3A & S3B). In total cell population, the basal transcript levels of LIFR in MCF7 and primary breast cancer cells were low when compared to MCF12A cells.(Figure 1F). Protein expression analyses for LIFR also revealed trends in all groups which were similar to the transcript data (Figure 1G and 1H). Analyses of human normal breast tissues and breast cancer tissues showed lower expression levels of LIFR at both the transcript and protein levels in breast cancer tissues compared to normal breast tissues (Figure S1B, S1C). These findings suggested an inverse correlation between miR-125a and LIFR in stem cell populations, but not in the bulk cell population.

To verify molecular interactions between miR-125a and LIFR, a standard 3′UTR luciferase reporter assay was performed. The 3′UTR of LIFR was split (3′UTR-1 and 3′UTR-2) and cloned into two separate plasmids with overlapping sequences. The plasmids were then transfected into HEK293 cells to generate two cell lines with stable expression of 3′UTR-1 or 3′UTR-2. There was an inhibition of luciferase activity by 45% in 3′UTR-1 with ectopic expression of miR-125a. We did not find any change in the luciferase activity of reporters fused with 3′UTR-2 and 3′UTR mutant (Figure 1H). Inhibition in luciferase reporter activity was an indication that there was an existing functional association between miR-125a and LIFR. We also performed the same experiment using MCF12A cells, and found similar luciferase activity inhibition (Figure S4). Together, the results indicated that LIFR was regulated by miR-125a.

### MiR-125a regulates stem cell pool dynamics

**Figure 2:**
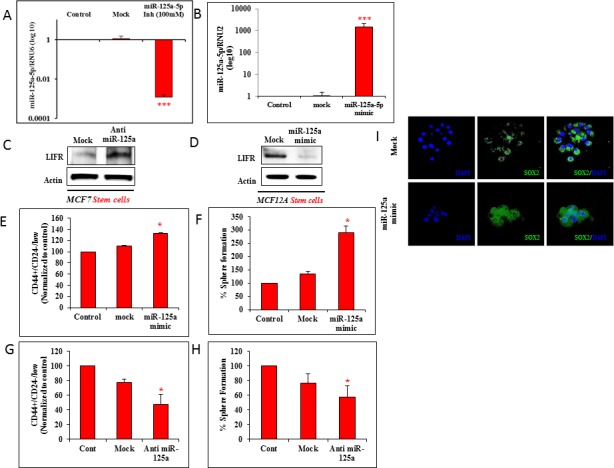
miR-125a modulation affects malignant and non-malignant breast epithelial stem cells **A.** qRT-PCR data shows effective inhibition of miR-125a in MCF7 cells using 100nM of antagomirs. **B.** qRT-PCR data shows effective over expression of miR-125a in MCF12A cells with miR-125a mimic. **C.** Immunoblotting analysis for LIFR post miR-125a inhibition in MCF7 CSCs. **D.** Immunoblotting analysis for LIFR in miR-125a over expressing MCF12A stem cells. **E.** Flow cytometric analysis shows increased percentage of stem cells with miR-125a over expression in MCF12A cells. **F.** Sphere forming assay demonstrates higher percentage of 3D sphere forming cells in miR-125a over expressing MCF12A. **G.** Flow cytometric analysis shows decreased percentage of stem cells with miR-125a inhibition in MCF7 cells. **H.** Sphere forming assay demonstrates lower percentage of 3D sphere forming cells in miR-125a inhibited MCF7 cells. **I.** Immunostaining of MCF12A spheres for SOX2 expression with over expression of miR-125a compared to mock controls.

To assess the impact of miR-125a and LIFR interactions on stem cell populations, MCF12A cells were treated with the miR-125a mimics and MCF7 cells were treated with the miR-125a antagomirs. Effective miR-125a inhibition in MCF7 cells, and overexpression in MCF12A cells were achieved in 24 hrs (Figure 2A and 2B). Reduced expression of miR-125a in MCF7 cells dramatically increased LIFR protein expression (Figure 2C). In contrast, LIFR protein expression in miR-125a overexpressing MCF12A cells (Figure 2D) showed the opposite trend of decreased LIFR protein expression. These findings provided further evidence that miR-125a regulated the expression of LIFR.

Changes in CD44^+^/CD24^−/low^ stem cell populations at 24 hrs after miR-125a modulation were then determined. Percentage of stem cells was observed to be increased by 22.15% with miR-125a overexpression in MCF12A cells, when compared to mock treated control cells (Figure 2E). These results were further supported by data obtained from sphere forming assays. Increased ectopic levels of miR-125a enhanced the sphere forming ability (156.51%) of MCF12A cells when compared to mock treated control cells (Figure 2F). However, miR-125a inhibition in MCF7 cells led to a decreased percentage (30.15%) of stem cells compared to mock control cells (Figure 2G). In addition, miR-125a inhibition also led to a decrease in sphere formation by 19.83% in MCF7 cells, compared to mock control cells (Figure 2H).

We also performed immunofluorescence for SOX2, a stemness protein marker in miR-125a overexpressing MCF12A spheres. Interestingly, miR-125a promoted the expression of SOX2 (Figure 2I), which implied that miR-125a enhanced stemness in these cells. Overall, these findings suggested a regulatory role for miR-125a in nonmalignant and malignant breast epithelial stem cells.

### LIFR modulation mimicked the effects of miR-125a on breast epithelial stem cells

**Figure 3:**
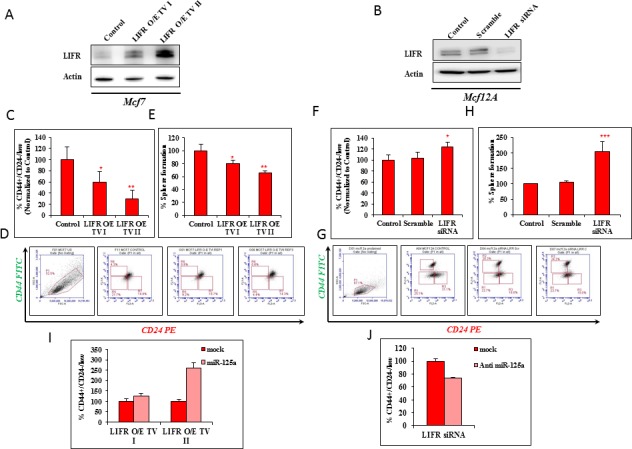
LIFR modulation mimics the effects of miR-125a **A.** Immunoblotting analysis for expression of LIFR in MCF7 with stable over expression of LIFR. **B.** Immunoblotting analysis for expression of LIFR in MCF12A (MCF12A-siLIFR) with transiently silenced LIFR. **C.** & **D.** Flow cytometric analysis demonstrating decrease in the percentage of CSCs in LIFR over expressing MCF7. **E.** Sphere forming assay shows decrease in the percentage of cells capable of forming 3D spheres with over expression of LIFR. **F.** & **G.** Flow cytometric analysis demonstrating increase in the percentage of CSCs in LIFR silenced MCF12A cells. **H.** Sphere forming assay shows increase in the percentage of cells capable of forming 3D spheres with LIFR silencing. **I.** miR-125a overrides the effects of LIFR over expression in MCF7 cells as evident by the increased percentage of CD44^+^/CD24^−/low^ cells. **J.** Inhibition of miR-125a abolishes the effect of LIFR silencing by reducing the percentage of CD44^+^/CD24^−/low^ cells.

We next determined if the regulatory effects of miR-125a were mediated through LIFR. Two transcript variants of LIFR (NM_002310.5 and NM_001127671.1) were stably overexpressed and designated as TV I and II (transcript variant I and II). A stable MCF7 cell line with LIFR (MCF7-LIFR) overexpression was successfully established (Figure 3A). We also performed transient inhibition of the expression of LIFR in MCF12A cells (MCF12A-siLIFR) using RNA interference (Figure 3B). The stem cell population (CD44^+^/CD24^−/low^) was significantly decreased in MCF7-LIFR TVI and MCF7-LIFR TVII cells by 41.01 and 70.34%, respectively (Figure 3C and 3D). The decrease in sphere forming ability (by 19.72 and 34.51% in MCF7-LIFR TV I and TV II cells, respectively) compared to control cells was reflective of the decreased percentage of stem cells after LIFR overexpression in malignant breast epithelial cells (Figure 3E). However, there was a notable increase in the CD44^+^/CD24^−/low^ population, by 20.17% in MCF12A-siLIFR cells (Figure 3F and 3G), as measured by flow cytometry. This was comparable to the effect of miR-125a overexpression on MCF12A cells. The data using MCF12A-siLIFR cells also demonstrated an increase in the percentage of sphere forming cells by approximately 100% as compared to scrambled control-treated cells (Figure 3H). This data supported the flow cytometry data.

To further validate the effect of miR-125a on stem cell pool homeostasis, we used miR-125a mimics in MCF7-LIFR cells and checked for the percentage of CD44^+^/CD24^−/low^ cells. MiR-125a was able to override the effects of MCF7-LIFR TVI and II by favoring expansion of the stem cell pool (Figure 3I). Similarly, in case of MCF12A-siLIFR, inhibition of miR-125a partially reduced the percentage of CD44^+^/CD24^−/low^ cells (Figure 3J). Overall, the results suggested that miR-125a regulates stem cell pool homeostasis by regulating LIFR.

### MiR-125a regulated breast epithelial stem cells through Hippo signaling downstream of LIFR

**Figure 4:**
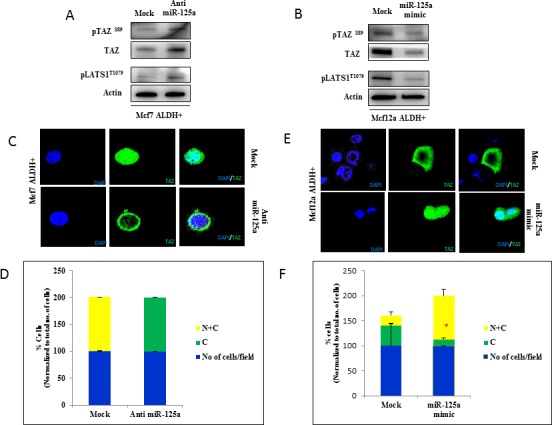
miR-125a impacts activity of Hippo signaling pathway through LIFR **A.** Immunoblotting analysis in MCF7 stem cells showing the increased activity of LATS1 and TAZ after inhibition of miR-125a. **B.** Immunoblotting analysis in MCF12A stem cells showing the decreased activity of LATS1 and TAZ with over expression of miR-125a. **C.** & **D.** Immunostaining for TAZ in MCF7 stem cells demonstrates sequestration of TAZ in the cytoplasm compared to mock control after miR-125a inhibition. The bar diagram shows percentage of cells with cytoplasmic or cytoplasmic and nuclear sub cellular localization of TAZ. **E.** & **F.** Immunostaining for TAZ in MCF12A stem cells demonstrates nuclear localization of TAZ with over expression of miR-125a compared to mock control. The bar diagram shows percentage of cells with cytoplasmic or cytoplasmic and nuclear sub cellular localization of TAZ.

LIFR is an upstream regulator of the Hippo signaling pathway [6]. We hypothesized that miR-125a regulated breast epithelial stem cells through the Hippo signaling pathway mediated by LIFR. In this current study, we investigated the activity of Hippo signaling in response to miR-125a modulation in breast epithelial stem cells. We analyzed the phosphorylation status of key proteins involved in Hippo signaling, along with subcellular localization of TAZ after miR-125a modulation in breast epithelial stem cells. We found that miR-125a inhibition in MCF7 stem cells led to activation of Hippo signaling. Phosphorylation of LATS1 (T1079), and TAZ (Ser89) in miR-125a inhibited MCF7 stem cells was increased compared to mock control (Figure 4A). In contrast, phosphorylation of LATS1 and TAZ was inhibited with miR-125a overexpression in MCF12A stem cells (Figure 4B). In addition, we also used immunofluorescence to determine the subcellular localization of TAZ. Cytoplasmic sequestration of TAZ in miR-125a inhibited MCF7 stem cells compared to mock control cells (Figure 4C and 4D) was observed. MiR-125a overexpressing MCF12A cells showed an increased nuclear localization of TAZ compared to mock control cells (Figure 4E and 4F).

**Figure 5:**
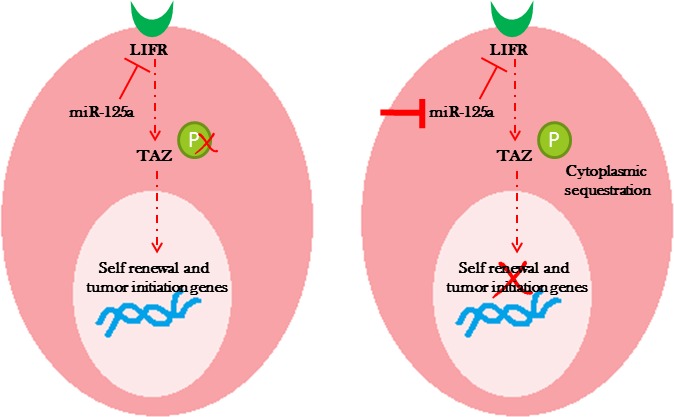
Diagram represents a proposed model of miR-125a regulation of TAZ through LIFR leading to changes in the stem cell pool homeostasis

These findings suggested that inhibition of miR-125a promoted phosphorylation of TAZ, resulting in its cytoplasmic sequestration. Cytoplasmic sequestration of TAZ could negatively affect the expression of stem cell self-renewal signaling networks, suggesting that miR-125a regulated the stem cell pool homeostasis by influencing the Hippo signaling pathway (Figure 5).

### MiR-125a suppression in primary breast cancer cells led to reduction in CSC traits

**Figure 6:**
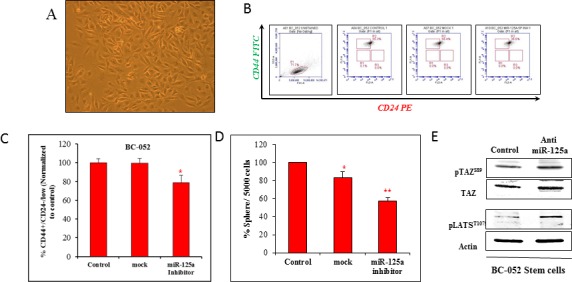
Targeting miR-125a in primary breast cancer cells **A.** Micrograph of primary breast cancer cells in culture **B.** & **C.** Flow cytometric analysis showing the decreased percentage of CSCs with inhibition of miR-125a **D.** Sphere forming assay demonstrating a decreased capacity of cells to form 3D spheres after inhibition of miR-125a **E.** Immunoblotting analysis showing increased activity of LATS and TAZ in CSCs after inhibition of miR-125a.

Our aim was to validate the findings obtained from miR-125a modulated malignant and non-malignant breast epithelial cell lines. Therefore, we used human primary breast cancer cells, BC052 (Figure 6A) and BC051 (Figure S5A, B & C). The expression of miR-125a in CSCs (ALDH+) derived from human primary breast cancer cells were compared to non-stem cells. The results indicated an increase in the expression of miR-125a in CSCs by approximately 9 fold when compared to non-stem cancer cells (Figure S4A). Suppression of miR-125a in these CSCs led to a decrease in the CD44^+^/CD24^−/low^ cell population by 20.60% compared to mock treated control cells in both cell lines (Figure 6B, 6C, and Figure S4B, C). Sphere forming assays revealed a reduction in the sphere forming capacity by 25.54% compared to mock control cells (Figure 6D). Based on these data, it was clear that miR-125a was capable of influencing the CSC population in primary breast cancer tissues.

### MiR-125a suppression activated Hippo signaling in CSCs

To further validate the proposed mechanism of miR-125a action on CSCs, immunoblotting for key proteins of Hippo signaling was performed. Notably, miR-125a inhibition promoted the activation of Hippo signaling, with increased phosphorylation of LATS1 and TAZ (Figure 6E). These findings in patient-derived breast cancer cells clearly confirmed the data obtained from cell lines.

### Effect of LIF on the action of miR-125a

**Figure 7:**
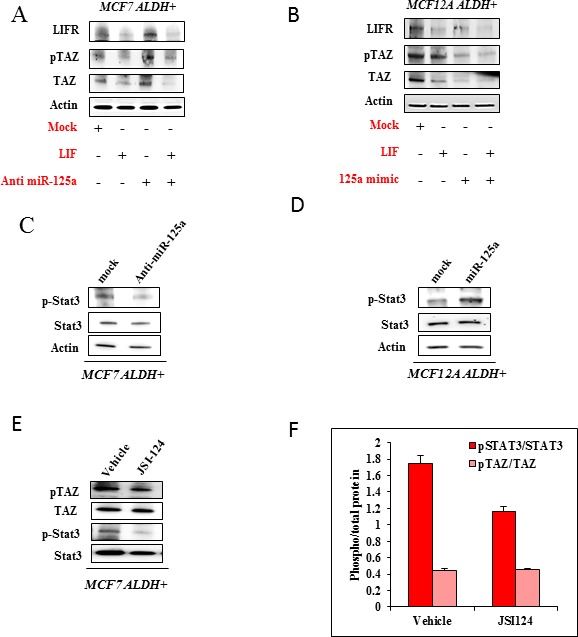
Effect of miR-125a on JAK2-STAT3 regulation by LIF-LIFR interaction **A.** & **B.** Immunoblotting analysis for MCF7 and MCF12A stem cells, demonstrating the influence of LIF and miR-125a inhibitor/mimic on TAZ. **C.** & **D.** Immunoblotting analysis showing the expression of pSTAT3/STAT3 in MCF7 and MCF12A stem cells when challenged with miR-125a inhibitor and mimic respectively. **E.** Immunoblotting analysis showing the expression of pTAZ/TAZ when treated with JAK2-STAT3 pathway inhibitor, JSI-124 in MCF7 stem cells. **F.** Bar diagram depicting the phospho/total forms of STAT3 and TAZ corresponding to immunoblots in E.

As we show that miR-125a acts through LIFR, it was imperative to understand the role of its ligand LIF on the functioning of Hippo signaling (Figure 7A and 7B). With addition of LIF, there was a reduced expression of LIFR in both MCF7 and MCF12A stem cells. Further, we observed that LIF favors inactivation of Hippo signaling which is evident by the decreased levels of phosphorylated TAZ; although the effect was more pronounced in MCF7 stem cells. Addition of LIF in combination with miR-125a inhibitor in MCF7 stem cells increased the levels of phosphorylated TAZ but didn't alter the levels of LIFR as compared to the LIF only group. We believe suppression of miR-125a in MCF7 stem cells is able to rescue the activation of Hippo signaling to some extent even in the presence of LIF. Addition of LIF in combination with miR-125a mimics in MCF12A stem cells decreased the levels of phosphorylated TAZ along with LIFR. This data demonstrates that addition of LIF along with miR-125a has an additive effect for inactivation of Hippo signaling in MCF12A stem cells.

### Influence of miR-125a on JAK2-STAT3 signaling pathway

The regulation of miR-125a on Hippo signaling through LIFR prompted us to investigate the effect of miR-125a modulation on the activation of STAT3, since JAK2-STAT3 is the classical downstream signaling pathway for LIF-LIFR interaction. We observed that miR-125a increased the phosphorylation of STAT3 in MCF12A derived stem cells and inhibition of miR-125a led to decreased phosphorylated STAT3 in MCF7 derived stem cells (Figure 7C and 7D). In this context, it appears that miR-125a activates STAT3 by inhibiting Hippo signaling. To further investigate possible cross talk between JAK2-STAT3 and Hippo signaling, we used JAK2-STAT3 inhibitor, JSI124 in MCF7 derived stem cells. Inhibition of JAK2-STAT3 signaling pathway doesn't appear to alter phosphorylation of TAZ (Figure 7E and 7F). In lieu of the current findings, Hippo signaling seems to have an influence on JAK2-STAT3 pathway. Moreover, JAK2-STAT3 inhibition doesn't seem to influence Hippo signaling. Preliminary evidence from our findings indicates that JAK2-STAT3 may be functioning downstream of Hippo signaling, but further experiments are warranted in this regard.

## DISCUSSION

CSCs are unique cellular components of the cancer microenvironment, with the greatest potential to lead to secondary cancers or metastasis [1]. MicroRNAs are key repressors of genes in cancer and can function as oncogenes or tumor suppressors [10]. Recently, various microRNAs have been identified to be aberrantly expressed in breast cancer [11]. In general, the anticancer or pro-cancer effects of microRNAs are assumed through their expression in general cancer cell population. However, microRNAs have an environment-dependent expression pattern, and expression in the general cancer cell population, unlike in CSCs, might not accurately represent expression in a minority cell population. In this study, we examined the role of miR-125a in the regulation of malignant (CSC) and nonmalignant breast epithelial stem cells.

Our findings demonstrated that miR-125a is tumor suppressor microRNA in bulk tumor cells of breast cancer origin, in agreement with previous studies [12-14]. However, miR-125a is a cancer promoting microRNA in breast epithelial stem cells, unlike its action as a tumor suppressor in breast cancer cells. This suggested that miR-125a played a different role in stem cells than in the bulk cell population.

The current study suggested that expression levels of miR-125a affects stem cell pool homeostasis in both malignant and nonmalignant conditions. Overexpression or inhibition of miR-125a enhanced or suppressed stem cell expansion, respectively. These findings were in agreement with previous reports regarding the role of miR-125a in stem cells of hematopoietic malignancies [15, 16]. Therefore, it is clear that miR-125a has a different role in stem cells compared to the remaining cell population under malignant and nonmalignant conditions.

In this report, we identified LIFR as one of the targets for miR-125a. LIFR is a known tumor/metastatic suppressor [6] for breast cancer and its downregulation in breast cancer has been demonstrated [17-19]. In agreement with previous studies [9], our results also showed a significant downregulation of LIFR in breast cancer cells compared to nonmalignant breast epithelial cells. We also showed miR-125a modulation leads to alterations in LIFR expression in malignant and nonmalignant breast epithelial stem cells. Increase in miR-125a significantly decreased LIFR in nonmalignant epithelial stem cells. An inverse trend was observed with inhibition of miR-125a in CSCs. Furthermore, our data demonstrated that suppression of LIFR in nonmalignant breast epithelial cells resulted in an increased stem cell percentage, and an inverse effect was observed in LIFR overexpressing breast cancer epithelial cells.

We further demonstrated that miR-125a influenced the Hippo signaling pathway through LIFR. Hippo signaling is a known tumor suppressor pathway [20-21] and inhibition of miR-125a in CSCs leads to its activation. In nonmalignant breast epithelial stem cells, overexpression of miR-125a inhibited the Hippo signaling pathway. Moreover, our findings provided evidence for altered subcellular localization of TAZ, after miR-125a modulation, which can be linked to activation/inactivation of Hippo signaling. LIFR has recently been shown to be an upstream regulator of Hippo signaling [6]. The miR-125a acts upstream of LIFR, thereby influencing the Hippo signaling pathway, downstream pro-proliferative and stem cell self-renewal genes, and finally the stem cell pool. It partially regulated the switch responsible for stem cell pool expansion or reduction, which significantly impacts breast epithelial stem cells in malignant and nonmalignant states.

It is evident that addition of LIF influences Hippo signaling in a negative manner. But addition of LIF in miR-125a suppressed MCF7 stem cells, actually rescues the activation of Hippo signaling to some extent. On the other hand, ectopic expression of miR-125a in LIF treated MCF12A stem cells inactivate Hippo signaling. Most of the available literature suggests a role for LIF in regulation of YAP; a transcriptional coactivator of Hippo signaling which has a significant homology with TAZ (6, 22-23). Ian Lian *et al* and Tamm C *et al* have shown that LIF withdrawal in embryonic stem cells cultures leads to increased phosphorylation of YAP. However, Chen *et al.,* have shown contrasting results demonstrating enhanced pYAP with addition of LIF in human breast cancer cell lines. Our results are somewhat in agreement with the studies from the embryonic stem cell cultures, and might provide an explanation about the different roles of LIF in different cellular context. Further, our findings reveal positive regulation of JAK2-STAT3 signaling by miR-125a. Inhibition of LIFR induced by miR-125a activated JAK2-STAT3 pathway stimulating a pro-carcinogenic molecular event in the non-malignant breast epithelial stem cells along with the inactivation of Hippo-TAZ signaling. LIF is known to canonically activate STAT3, and LIFR inhibition by miR-125a appears to mimic the molecular phenomenon in this context. Very little information is available about cross-talk between Hippo signaling and JAK2-STAT3. Our preliminary data suggests that JAK2-STAT3 signaling can be functioning downstream of Hippo signaling because the JAK2-STAT3 inhibition study demonstrated that the pTAZ levels in MCF7 stem cells are not affected, but inhibition of pTAZ levels by miR-125a negatively alters the pSTAT3 levels.

Overall, our findings strongly suggested that miR-125a is important for the proliferative fate of both malignant and nonmalignant breast epithelial stem cells, therefore further affecting various stages of carcinogenesis. To achieve better treatment response and prognosis, miR-125a could potentially be a target for use with conventional chemotherapy against CSCs. MiR-125a suppression could significantly control the CSC population and tumor suppression through LIFR, thus activating tumor suppression and the Hippo signaling pathway.

## MATERIALS AND METHODS

### Cell culture

Cell lines MCF7, MCF12A, MCF10A, BT-20, T47D, MDA-MB-231 and HEK293 were purchased from commercial sources (ATCC, Manassas, VA, USA). Breast cancer patient-derived primary cell lines, BC-051 and BC-052 were established from human breast tumor samples. MCF7, MDA-MB-231, T47D, BT-20 and primary breast cancer cells were maintained in RPMI and 10% fetal bovine serum (FBS). MCF12A cells were maintained in DMEM/F12 and 5% horse serum, using the SingleQuots™ (Lonza, Portsmouth, NH, USA; Cat No: CC-4133). HEK293 cells were maintained in MEM medium with 10% FBS. MCF10A cells were maintained in mammary epithelial basal media (MEBM) media supplemented with SingleQuots.

### Primary tissue collection

We collected breast cancer tissue from the patients and normal breast tissue from healthy volunteers. Protocols for obtaining patient consent to donate breast tissue and for the preservation of patient confidentiality (no patient identities were obtained) were reviewed and approved by the Institutional Review Board of Texas Tech University Health Science Center in El Paso, TX.

### Stem cell isolation

Stem cells were isolated by sorting aldehyde dehydrogenase bright (ALDH+) populations, using commercially available kits from Stem Cell Technologies (Vancouver, BC, Canada; ALDEFLUOR kit, Cat No. 01700). This enabled the selection of stem cells with strong aldehyde dehydrogenase (ALDH) activity. An inhibitor of ALDH was used as the negative control. The cells were analyzed on a flow cytometer in the green fluorescence channel (520–540 nm), and were then subjected to sorting. Sorting was performed using a flow cytometric cell sorter (FACS Aria; BD Biosciences, San Jose, CA, USA). ALDH positive cell sorting was performed for MCF7, MCF12A, MCF10A, BT-20, T47D, MDA-MB-231, BC051, and BC052 cells.

### *In silico* analysis

miR-125a binding site on LIFR 3′UTR was obtained using TargetScan online portal. (http://www.targetscan.org/;version 6.2).

### Transfections

Antagomirs (Qiagen, Valencia, CA, USA; Cat. No. MIN0000443) and mimics (Qiagen; Cat. No. MSY0000443) for miR-125a were used to modulate miR-125a expression. Transient transfections for the mimics and antagomirs were performed using HiPerfect Transfection reagent as per the manufacturer's protocol (Qiagen; Cat. No. 301707). One hundred nM and 10 nM of the miR-125a antagomirs and mimics were used for MCF7 and MCF12A, respectively. One hundred nM of the miR-125a antagomirs were also used for primary breast cancer cells.

For over expression of LIFR in MCF7, commercially available cDNA clones in pCMV6-Entry vector was obtained (Origene; Cat. No. RC226327 & RC220696). The two transcript variants of LIFR encode the same protein. Transfection was carried out using Lipofectamine 2000 (Invitrogen) and manufacturer's instructions were followed for transfection to generate stable LIFR over expressing MCF7 cell lines.

### Sphere formation assay

Sphere formation assays were established for MCF7, MCF12A, and BC-052 cells using 5000 cells/well on ultra-low attachment 6-well plates (Corning, Corning, NY, USA; Cat. No.3471). Cells were grown in serum free medium containing Dulbecco's modified eagle medium (DMEM)/F12+ GlutaMAX-1 or RPMI supplemented with 2% B27 (Gibco Life Technologies, Grand Island, NY, USA) and MEGM SingleQuots (hydrocortisone, insulin, beta-mercaptoethanol, EGF, and gentamycin) (Cat. No. CC-4133; Lonza). The plates were incubated for 5-7 days before analysis under a phase contrast light microscope (Nikon Eclipse TS100; Nikon, NY, USA).

### Flow cytometry

MCF12A, MCF7, BC-051, and BC-052 cells were cultured to 70-80% confluency and treated with miR-125a mimics/antagomirs. After 24 hrs of treatment, the cells were detached from the culture dishes using trypsin-EDTA. Cells (1 × 10^6^ cells for each group) were probed with CD44-FITC (BD; Cat. No. 555478) and CD24-PE (BD; Cat. No. 555428), after fixing and blocking with 5% formaldehyde and 0.1% bovine serum albumin (BSA), respectively. Samples were incubated for 30 min in the dark at room temperature, and finally washed with 1× phosphate buffered saline (PBS) and subjected to flow cytometric analyses for CD44 and CD24. Unstained and single stained compensation controls for CD44 and CD24 were used to determine gating for the analyses. Acquisition and analyses of 10,000 gated cells were accomplished using the BD Accuri™ C6 (BD, Cat. No.653118) for percentage determination of cells with the CD44^+^/CD24^−/low^ phenotype.

### Quantitative reverse transcriptase real time PCR (qRT-PCR)

Total RNA was extracted from the cells using TRIzol reagent (Life Technologies; Cat. No. 15596-018). cDNA for microRNA and gene expression analyses was prepared using the miScript II RT kit (Qiagen; Cat. No. 218161) and RT2 the first strand kit (Qiagen; Cat. No. 330401), respectively. The qRT-PCR was performed in triplicate using the miScript SYBR Green PCR kit (Qiagen; Cat. No. 218073) and the Quantitech SYBR green kit (Qiagen; Cat. No. 204141) on a StepOnePlus real time PCR system (Applied Biosystems, Foster City, CA, USA). The analyses were based on the comparative Ct method (2^−ΔΔCt^) with RNU6-2 and GAPDH or 18SRNA as the reference genes for microRNA and other genes, respectively. Human specific primers were purchased from Qiagen (Quantitech Primer Assays; miR-125a-5p; Cat. No. MS00003423, and RNU6-2; Cat. No. MS00033740), LIFR (Cat. No. QT00026873), 18SRNA (Cat. No. QT00199367) and GAPDH (Cat. No. QT00079247).

### Immunoblotting

Cell lysates were obtained using M-PER Mammalian Protein extraction reagent (Thermo Scientific, San Jose, CA, USA; Cat. No. 78501) and Halt Protease and Phosphatase Inhibitor Cocktail (Thermo Scientific; Cat. No. 1861281). Lysates were cleared by centrifugation and proteins were quantified by BCA Protein Assay (Pierce; Rockford, IL, USA; Cat. No.23225). Equal amounts of protein were separated by 4-20% gradient sodium dodecyl sulphate polyacrylamide gel electrophoresis (SDS-PAGE) gels and transferred onto PVDF membranes. Immunoblotting was performed, and antibodies specific for LIFR (Santa Cruz Biotechnology, Santa Cruz, CA, USA; Cat. No. sc-659), TAZ (Cell Signaling Technology, Danvers, MA, USA; Cat. No. 4883S), pTAZ (Santa Cruz Biotechnology; Cat. No. sc-17610), pLATS (Cell Signaling Technology; Cat. No. 9159S), and beta actin (Sigma-Aldrich, St. Louis, MO, USA; Cat. No. A1978) were detected using horseradish peroxidase (HRP) conjugated anti-mouse antibody (Santa Cruz Biotechnology; Cat. No. sc-2055), anti-rabbit (Santa Cruz Biotechnology; Cat. No. sc-2054), or anti-goat (Santa Cruz Biotechnology; Cat No: 2056) antibodies. For chemiluminescent detection of proteins, Super Signal West Femto Chemiluminescent Substrate (Thermo Scientific; Cat. No. 34095) was used according to the manufacturer's instructions. Immunoblots were processed digitally on the Image Quant LAS4000 Biomolecular Imager (GE Healthcare, Pittsburgh, PA, USA). The signal intensities for each antibody were densitometrically analyzed and normalized to actin bands.

### Luciferase reporter assay

For the luciferase assay, 3′UTR for LIFR was cloned into the pEZX-MT01 dual luciferase (firefly and renilla) vector (Genecopoeia, Rockville, MD, USA; Cat. No. HmiT010608). A control plasmid without the 3′UTR sequence was used as a negative control (GeneCopoeia; Cat. No. CmiT000001-MT01). Both were used to generate stable clones in HEK293 cell lines.

Stable clones of HEK293 expressing the 3′UTR or control sequences were plated at 60-70% confluency before they were transfected with 10 nM miR-125a mimic, using HiPerfect transfection reagent. Simultaneously, stable clones of HEK293 expressing the control plasmid were transfected with the miR-125a mimic. Cells were then used for determination of luciferase activity at 72 hrs. The Luc-Pair miR Luciferase Assay kit (GeneCopoeia; Cat. No. LPFR-M100) was used for detection of luciferase activity by use of the CLARIOStar® plate reader (BMG Labtechs, Hertfordshire, UK).

### Immunofluorescence

The sorted stem cells/spheres were plated onto poly lysine-coated, 8-well chambered slides with media containing 1% FBS, and incubated at 37°C with 5% CO_2_ for 3-4 hrs, for attachment. Cells were fixed using 5% formaldehyde and blocked with 5% BSA for 1 hr, and then stained with antibodies specific to TAZ and SOX2 (Santa Cruz Biotechnology; goat IgG clone Y-17, 1:100 dilution). Alexa Fluor 488- and 594-tagged secondary antibodies were raised as species appropriate for the primary antibody detection. The cells were washed and mounted after counterstaining with DAPI. All slides were examined using a confocal microscope (Nikon Eclipse Ti; Nikon, NY, USA). Multicolor images were collected sequentially in two/three channels.

### Statistical analyses

All experiments were performed for a minimum of three times. Student's *t*-test was used for all experiments. The statistical analyses were performed with Prism GraphPad software (La Jolla, CA, USA). A *P* < 0.05 was defined as statistically significant. All data are represented as means ± SEM.

## SUPPLEMENTARY MATERIAL FIGURES


