# Supplementary material for: MicroRNA-125a influences breast cancer stem cells by targeting leukemia inhibitory factor receptor which regulates the hippo signaling pathway
Source: Oncotarget. 2015 Apr 29;6(19):17366–78. doi: 10.18632/oncotarget.3953 (PMC4627314; doi:10.18632/oncotarget.3953)
Supplement: Supplementary file 1 [file oncotarget-06-17366-s001.pdf]

MicroRNA-125a influences breast cancer stem cells by targeting leukemia inhibitory factor receptor which regulates the hippo signaling pathway

Supplementary Material

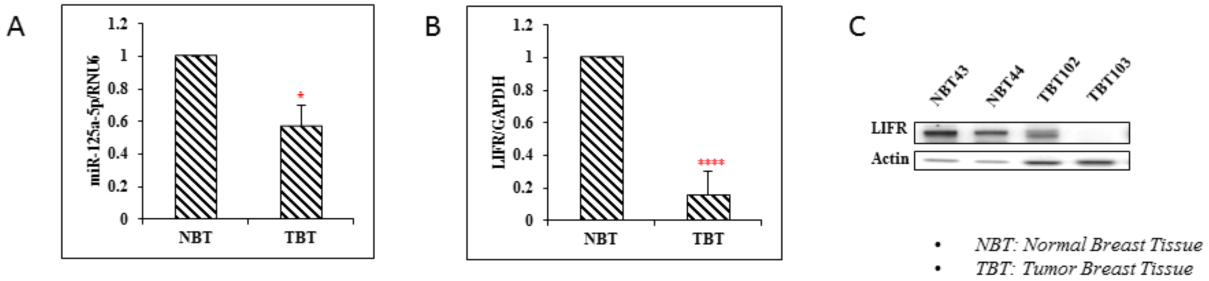

Supplementary Figure 1: (A) qRT-PCR data showing the expression of miR-125a in normal breast tissues (n=3) and tumor breast tissues (n=3). (B) Transcript level of LIFR in NBT and TBT samples. (C) Immunoblotting analysis of LIFR in normal breast tissues and tumor breast tissues.

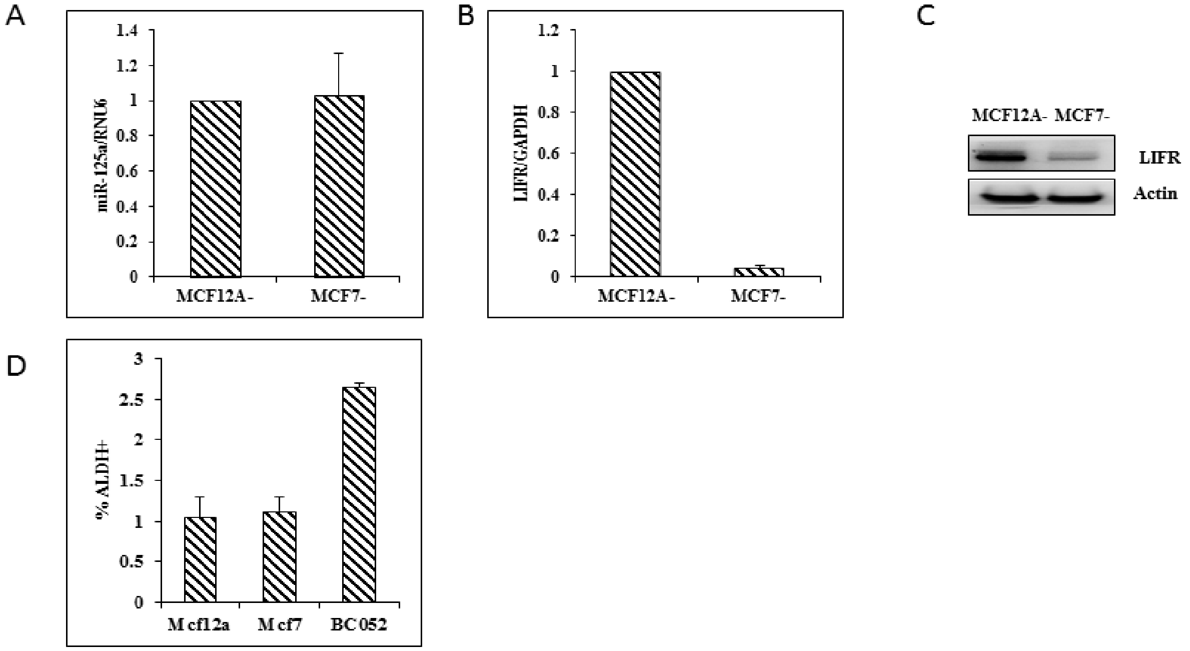

Supplementary Figure 2: (A) qRT-PCR data showing the expression of miR-125a in ALDH negative (ALDH-) population of MCF12A and MCF7 cells. (B) Transcript level expression of LIFR in ALDH- population of MCF12A and MCF7 cells. (C) Immunoblotting analysis of LIFR in ALDH- population of MCF12A and MCF7 cells. (D) Percentage of ALDH positive (ALDH+) cells in MCF12A, MCF7 and primary breast cancer cells.

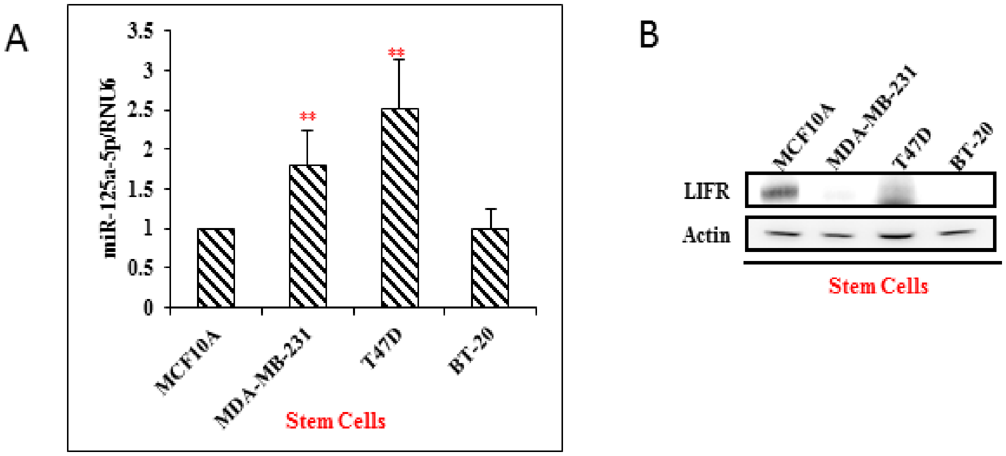

Supplementary Figure 3: (A) qRT-PCR analysis for miR-125a expression in stem cells derived from MCF10A, (B) Immunoblotting analysis for LIFR expression in cell lysates obtained from stem cells of MCF10A, MDA-MB-231, T47D and BT-20. Negative correlation between the expression of miR-125a and LIFR can be seen in all cell lines except BT-20.

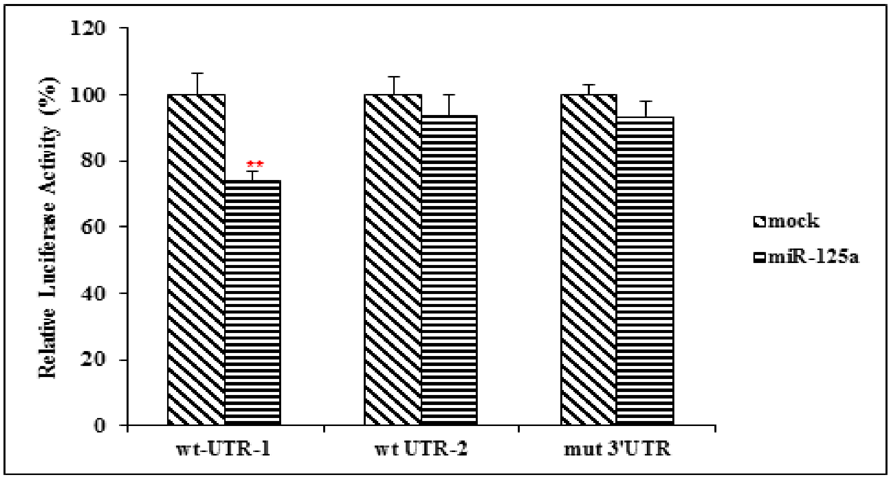

Supplementary Figure 4: Luciferase assay data for MCF12A cells demonstrates a decrease in the luciferase activity with addition of miR-125a mimics to LIFR 3'UTR expressing cells.

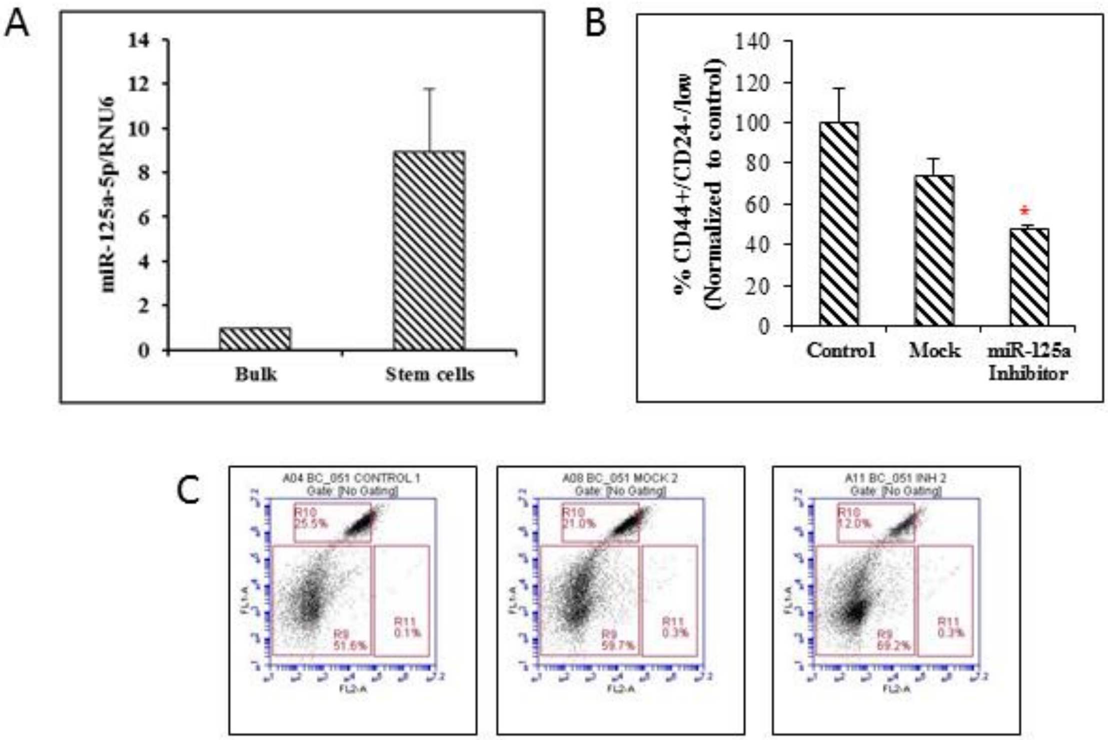

Supplementary Figure 5: (A) Expression levels of miR-125a in CSCs from primary breast cancer cells compared to the bulk tumor cells. (B & C) Inhibition of miR-125a in primary breast cancer cells (BC-051) leads to a decrease in the percentage of CSCs.
